# Innovations Developed by Patients and Informal Caregivers for Needs Associated to Rheumatic Diseases

**DOI:** 10.3389/fmed.2021.647388

**Published:** 2021-03-16

**Authors:** Maria João Jacinto, Pedro Oliveira, Helena Canhão

**Affiliations:** ^1^Patient Innovation, Lisbon, Portugal; ^2^Católica-Lisbon School of Business and Economics, Universidade Católica Portuguesa, Lisbon, Portugal; ^3^Copenhagen Business School, Copenhagen, Denmark; ^4^Nova School of Business and Economics, Universidade Nova de Lisboa, Lisbon, Portugal; ^5^CHRC, Comprehensive Health Research Center, NOVA Medical School, UNL, Lisbon, Portugal; ^6^EpiDoC Unit, Chronic Diseases Research Centre, Nova Medical School, Universidade Nova de Lisboa, Lisbon, Portugal; ^7^National School of Public Health, Universidade Nova de Lisboa, Lisbon, Portugal

**Keywords:** patient innovation, user innovation, health, rheumatic diseases, disability

## Abstract

Until recently, innovation in healthcare was mainly achieved through the development of new drugs, therapies, and medical devices by big pharma and medtech companies; however, the innovative potential for this field is much broader. The patients and caregivers' role in healthcare is often associated with disease management, demand for their own illness data, and its exchange with other patients. However, the patients and caregivers' capacity to innovate to cope with limitations associated with their health condition is a growing phenomenon and starting to be supported by healthcare stakeholders to achieve a truly patient-centric system. Our previous research has shown that these uncommon innovators can develop a wide range of solutions, from simple adaptations and products to highly technological biomedical devices. In this paper, we present novel solutions developed by rheumatic patients, their caregivers, and collaborators, published on the “Patient Innovation” platform (https://patient-innovation.com/), with a focus on the innovator profile, the need that triggers the innovative process, the type of motivation behind the product, and the products developed. The most significant needs that motivate innovation are the will to increase the level of independence (71%) and to be able to perform daily routine activities (65%). In over 80% of cases, the fact that the market does not fully fulfill the needs felt during daily activities is the main motivation to innovate. It is thus concluded that there is room for innovation in rheumatic diseases with solutions developed by patients and informal caregivers that intend to solve needs that the healthcare market is not covering.

## Introduction

Rheumatic diseases are in the group of diseases recognized earliest in the world (1), and although the most common rheumatic diseases present a high prevalence in the elderly population, rheumatic diseases affect people of all ages and currently have a great impact on patients' daily living as well as on society, as it is commonly reflected in high economic costs through a great consumption of healthcare and social resources.

Despite the critical importance of the healthcare sector for the economy and society, where average health spending reached 8.8% of gross domestic product (GDP) across the Organization for Economic Co-operation and Development (OECD) (2), current healthcare provision does not always meet patient's real and daily needs. To solve this, some patients and informal caregivers do not wait for a solution to come up in the market, and thus start developing innovative solutions to overcome limitations associated with the health condition that they face (3, 4). Von Hippel started to introduce the concept of user innovation as individuals or firms who expect to directly benefit from a product that they have created (5, 6); this concept differs from producer innovators—firms or individuals who develop novel products to benefit from selling it as a better or new product or service. Thus, when translating the concept of user innovation to the healthcare domain, patient innovators are citizens who develop useful and innovative solutions to cope with their health disorders. These patient innovations range from simple products for everyday use to unknown therapies and high-tech solutions (7). These findings suggested that patients and informal caregivers worldwide may contribute with a high number of innovative solutions with a valid need-based motivation and, if shared and adopted, can improve not only their quality of life but also the life of others with similar health conditions (8). This was the motivation for the creation of the website www.patient-innovation.com, a non-profit international, multilingual, and open platform which currently presents over 1,500 solutions developed by patients, informal caregivers, and collaborators which have been submitted or collected, medically validated, and shared.

In this paper, it is our intention to present and describe relevant innovative solutions developed by rheumatic patients, their informal caregivers, and collaborators, who decided to solve some of their everyday needs—originated by the health condition that they face—but could not find any solution in the market.

## Methods

In order to analyze innovative solutions developed by patients and informal caregivers, the ‘*Patient Innovation*’ platform (www.patient-innovation.com) was used as its presents itself as a centralized inventory of patient-developed solutions.

The ‘*Patient Innovation*’ platform provides the opportunity to patients and caregivers to actively engage in sharing solutions within the *Patient Innovation* community. In order to share a novel solution, the user must register on the website (the connection to *Patient Innovation* is always encrypted and personal data is only stored in high security data centers located in the European Union) and, once logged in, the user can write down his/her solutions for dealing with a condition in his/her daily life (information required: solution title, description, about the innovator, and optional images and videos). Before being published, the *Patient Innovation*'s medical team evaluates and validates (or not) the submitted solutions, by only approving the ones that are not drugs, chemicals, intake/topical substances, invasive devices, or other visibly and intrinsically dangerous proposals. Once these are online, they are available to everyone who may be interested, or can benefit from the published solutions.

The medically validated and published solutions in the ‘*Patient Innovation*’ platform were screened, and solutions that showed features to be possible solutions for rheumatologic diseases were selected. In a first stage analysis, disease-based motivation was screened and rheumatology-based solutions were distinguished from solutions that cover rheumatologic patients' needs/symptoms but were based on other medical conditions. Through the innovation description and information available about the innovator, the type of innovator (patient, caregiver, or collaborator), sex, country, product category (the exact role/goal of the solution developed, divided into the following categories: activities of daily living, pain/therapy, hobbies, and movement), motivation (why the innovators had to develop a novel solution to cope with a limitation/need related with the health condition that they face, divided into the following categories: “the products in the market were not useful to satisfy the need,” “there was not any alternative in the market,” “cost of the alternatives in the market,” and “other”) and need to innovate (the need that made the patient, caregiver, or collaborator to innovate and create a novel product, divided into the following categories: “to be able to perform personal hygiene tasks,” “to be able to perform a daily routine activity,” “to increase the level of independency,” “to be able to move,” “to reduce pain,” “to replace a lost function,” “to improve therapy,” and “other”) were studied. The criteria used for categorization was developed by the authors based on the information available in the solutions description fields (solution title, description, about the innovator, optional images, and videos) posted on the ‘*Patient Innovation*’ platform.

## Results

The ‘*Patient Innovation*’ platform presented 1,399 medically screened and published solutions when consulted on July 8^th^ 2020 for the purpose of this research work. From those, 101 published innovations showed features that marked them as possible solutions for rheumatologic patients; most of those solutions were developed for activities of daily living (54%), whereas for hobbies (24%), pain/therapy (15%), and movement (9%) are other significant types of products described in the platform.

Thirty-four percent (*n* = 34, Table 1) of those solutions were developed as a response to rheumatologic-based needs by patients, informal caregivers, and collaborators who face difficulties associated with this disease category. The great majority of rheumatologic-based solutions (82%) were developed in the same proportion by patients (those who develop solutions for themselves) and informal caregivers (those who develop solutions for loved ones and/or family); eighteen percent (*n* = 6) of the solutions were developed by collaborators (those who develop solutions for someone out of his/her family/friends circle). Fifty-four percent (*n* = 19) of those solutions were developed by men (46% by women) and by citizens from countries of five continents (e.g., USA, Portugal, China, Australia, and Kenya).

**Table 1:** Innovative solutions to cope with rheumatic diseases limitations developed by patients, informal caregivers, and collaborators published in https://patient-innovation.com/.

**Product**	**Type of innovator**	**Country of origin**	**Rheumatologic Disease**
Hair dryer adaptation for stiff upper limbs^1^	Patient	Portugal	Fibromyalgia
Home-made orthotic for foot pain^2^		USA	Arthritis
Home-made pouches for hot-cold therapy to reduce pain^3^		Canada	Arthritis
ViEx, a string-based device to reduce hand joints pain^4^		USA	Rheumatoid arthritis
Handmade toys to cope with Arthritis^5^		USA	Juvenile Arthritis
Food cutting board and knives to overcome poor hand grip^6^		New Zealand	Rheumatoid Arthritis
Tool to help with gardening without having to bend down^7^		Belgium	Arthritis
Adapted transport container for disabled people scooters^8^		Belgium	Ehlers-Danlos Syndrome
Nose-pad, a device to work in a computer/tablet using nose and lips^9^		Sweden	Arthritis
Gehrad, a walking aid device^10^		Germany	Osteoarthritis
Protective capes for wheelchair users^11^		Belgium	Ehlers-Danlos Syndrome
Sandi Gloves, an easier way to sand^12^		Australia	Arthritis
Chronically Simple, an app for chronic diseases management^13^		Canada	Ehlers-Danlos Syndrome
Fashionable walking aids^14^		UK	Fibromyalgia
Adaptor for car key turning aid to avoid wrist twisting motion^15^	Caregiver	USA	Arthritis
Accessible zipper pull^16^		USA	Arthritis
Assistive door locker^17^		USA	Arthritis
Blanket support to avoid overweighting joints and still not get cold^18^		Portugal	Arthritis
Light intensity regulator for car traveling ^19^		Portugal	Arthritis
Exoskeleton glove to enhance hand movements^20^		Greece	Arthritis
Wheelchair tank to ease beach and countryside visits^21^		UK	Arthritis
Egg collector to avoid having to bend over^22^		Belgium	Myopathy
Stair-climbing wheelchair^23^		China	Arthritis
Adapted clothes to enable minimum body movement while dressing^24^		China	Osteoarthritis
Stair-climbing booster^25^		China	Arthritis
Toothpaste squeezer for people with poor grip^26^		USA	Arthritis
Orthorod, an adapted fishing pole^27^		USA	Arthritis
Swachchta Suvidha Brush, a simple slip-on floor cleaner^28^		India	Spondylosis, Arthritis
Book page turning device^29^	Collaborator	USA	Arthritis
Kitchen safety handle for limited hand strength and flexibility^30^		Taiwan	Rheumatoid Arthritis
Obi, a robotic device for autonomous feeding^31^		USA	Arthrogryposis
3D printing device to help to dress^32^		USA	Arthrogryposis Multiplex Congenita
E-Con, an all-terrain wheelchair^33^		Kenya	Arthritis
Finger support to ease finger deformities^34^		USA	Arthritis

Patient Innovation platform location (https://patient-innovation.com/xxxx/zzzz):

1 */node/502*,

2 */post/654*,

3 */post/920*,

4 */post/1203*,

5 */post/1234*,

6 */post/1345*,

7 */post/1365*,

8 */post/1372*,

9 */post/1376*,

10 */post/1642*,

11 */post/1648*,

12 */post/1944*,

13 */post/2416*,

14 */post/2473*,

15 */post/587*,

16 */post/669*,

17 */post/670*,

18 */post/880*,

19 */post/881*,

20 */post/1204*,

21 */post/1266*,

22 */post/1362*,

23 */post/1843*,

24 */post/1845*,

25 */post/1849*,

26 */post/1901*,

27 */post/2088*,

28 */post/2355*,

29 */post/861*,

30 */post/1304*,

31 */post/1537*,

32 */post/2022*,

33 */post/2039*,

34 */post/2209*.

**Figure 1:**
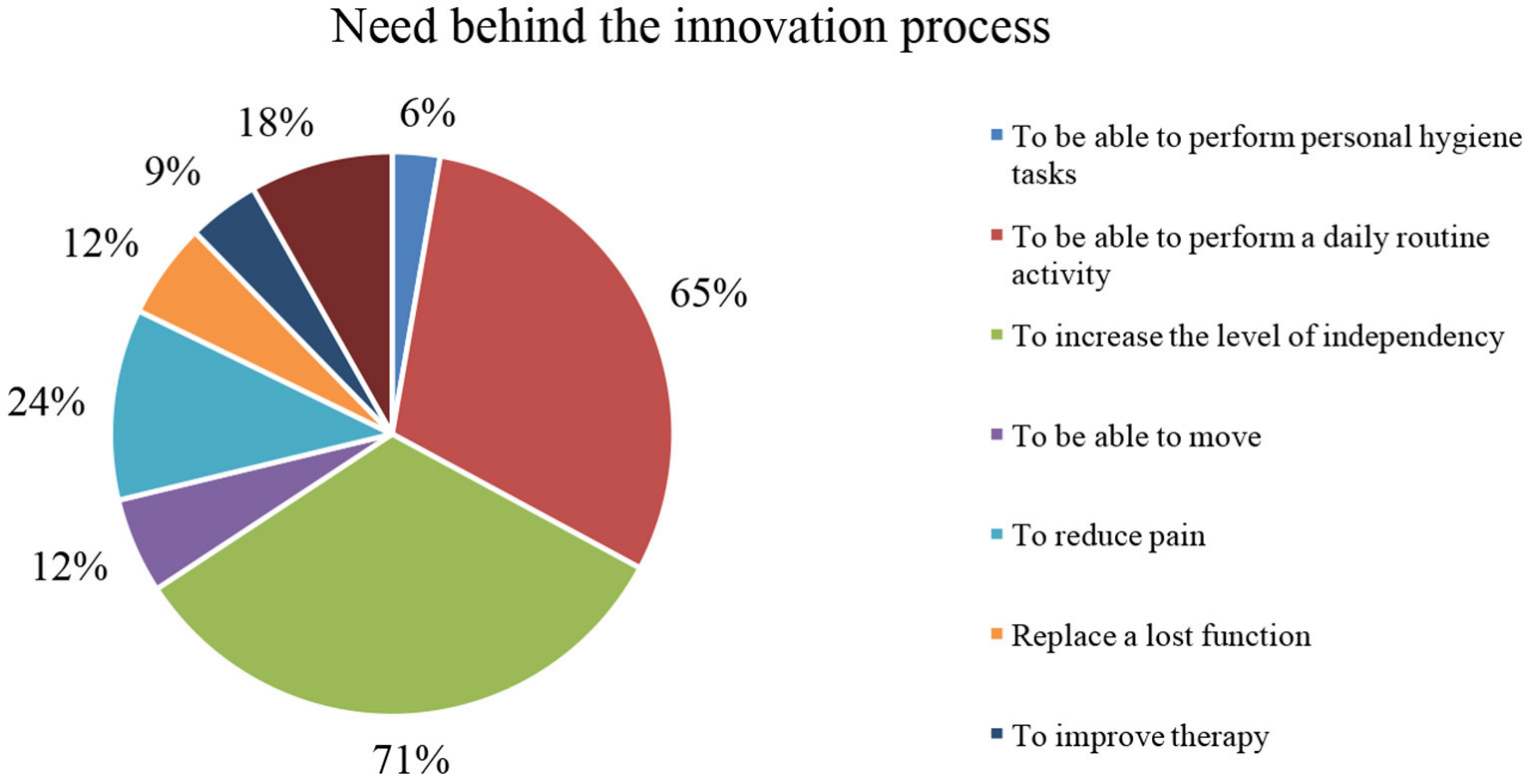
Need behind the innovation process of rheumatologic-based innovators.

When analyzing the need behind the innovation process for the rheumatologic-based solutions (Figure 1), the most significant ones rely on efforts to increase the level of independency (71%) and to be able to perform a daily routine activity (65%). Also, pain reduction (24%), replacing a lost function (12%), and mobility (12%) are other relevant needs found in the innovations analyzed.

Following the World Health Organization's definition for medical device (9), 32% of the solutions developed as a response to rheumatologic-based needs under analysis are framed as Class I medical devices (10), which meets the amount of pain reduction and replace a lost function as described above; still, 68% of the analyzed solutions are not framed as medical devices. However, only 15% of these rheumatologic-based solutions are approved by regulatory authorities and are currently being commercialized.

Consequently, the type of products developed by these uncommon innovators mainly rely on activities of daily living (41%), pain/therapy (24%), movement (18%), and hobbies (18%). These innovators found as their main motivation to innovate the fact that the products in the market did not fully meet a need they had (82%). Furthermore, the cost of alternative products (27%) and the inexistence of useful alternatives (9%) available in the market are also important motivations to these innovators to start developing novel solutions to cope with limitations that they face every day.

## Discussion

Although “innovation” is a broad term and it can be applicable in different disease's aspects, we can highlight that in the recent years “innovation” in rheumatology has widely expanded in digital health, namely through electronic health records, virtual visits, wearable technologies, and digital therapeutics which improve access, outcomes, adherence, and research (11, 12) or new therapeutic production approaches such as biosimilars (13). Still, this paper intends to expose that there is still some room for innovation through solutions developed by patients and informal caregivers who intend to solve needs that the healthcare market is not yet covering. To do that, the authors have analyzed 34 rheumatologic-based solutions published on *Patient Innovation*'s database, 14 developed by patients and 14 by informal caregivers, which reinforces that one major source of innovation are the needs that these citizens feel in their daily lives. The fact that 54% of those solutions were developed by men (46% by women) and by citizens from countries of five continents (e.g., USA, Portugal, China, Australia, and Kenya) reflects the patient innovation process to be as global and diverse as the medical diseases themselves. The products analyzed vary from simple devices (e.g., food cutting board and knives to overcome poor hand grip) to high-tech solutions (e.g., exoskeleton glove to enhance hand movements).

Thirty-two percent of the solutions developed under analysis are framed as Class I medical devices; however, only 15% are approved by regulatory authorities and are currently being commercialized, as most of the solutions here described were developed to solve a daily life need felt by patients and informal caregivers and were not manufactured on a large scale for commercialization (at least at the analysis moment). This is a common approach for patient innovators, as this kind of uncommon inventors have only developed their novel solutions because they have a validated need that motivated them to innovate, and thus their main goal was to fulfill that need, not to profit from it. Still, there has been an increasing number of patients and informal caregivers who have been establishing their own solutions in the market as an effective way to distribute their solutions to others with similar needs (14, 15).

The authors would like to highlight the potential of platforms such as ‘*Patient Innovation*’ and other information and communication technologies to promote an easy interaction between common citizens and healthcare professionals. Mainly, healthcare professionals and other stakeholders can use these resources to learn more about patients' real needs and the solutions that they have developed to cope with them, as well as to start including patients as consultants and their innovation process into the producer innovation processes. As innovation by patients and caregivers is still a new topic that has only started to be explored in recent years, it is important that healthcare industry representatives increase their awareness of this phenomenon and support this innovation process to achieve a more effective and cheaper product development process.

Although this work only shows a brief sample of novel solutions developed by common citizens for needs associated with rheumatic diseases, as the authors are sure that many more solutions exist developed by these uncommon inventors than the ones published in ‘*Patient Innovation*’ platform, it is thus concluded that solutions developed by patients and informal caregivers can significantly contribute to rheumatic diseases innovations as it aims to solve needs that the healthcare market is not yet covering.

## Data Availability Statement

The datasets presented in this study can be found in online repositories. The names of the repository/repositories and accession number(s) can be found below: https://patient-innovation.com/.

## Author Contributions

MJ was responsible for conceptualization, information extraction, formal analysis, methodology, writing, and edition. PO and HC were responsible for funding acquisition, project administration, resources, supervision, validation, writing, review, and editing. All authors contributed to the article and approved the submitted version.

## Conflict of Interest

The authors declare that the research was conducted in the absence of any commercial or financial relationships that could be construed as a potential conflict of interest.
